# Pulmonary Diseases of Elevator Employees

**Published:** 1879-12

**Authors:** Thomas F. Rochester

**Affiliations:** Professor of Principles and Practice of Medicine, Medical Department University of Buffalo


### THE BUFFALO MEDICAL AND SURGICAL JOURNAL.

Vol. XIX.—DECEMBER, 1879.—No. 5.

### Original Communications.

### PULMONARY DISEASES OF ELEVATOR EMPLOYEES.

BY THOS. F. ROCHESTER, M. D.,*

Professor of Principles and Practice of Medicine, Medical Department University of Buffalo.

In all great industries, whether commercial or manufacturing, there exists, of unavoidable necessity a condition of life or mode of living almost sure to develop a peculiarity and class of disease, incident to the labor of the employees. This is especially noticeable in all mining enterprises—in workers in metals, and in the operatives of cotton and woolen mills—as also in those of flouring mills, and they have formed the subject of many interesting papers; but the writer is not aware that the case of the "scooper" has ever received more than passing notice in the discussions of this and other medical societies. In order to present the matter properly, it is necessary to premise that there are twenty-five elevators, and five floating elevators in this city. The former employ from fifty to one hundred men

* Read before Buffalo Medical Association, Nov. 5, 1879.

each; the latter from fifteen to twenty-five, each. It is probably a low estimate to state the total number of "shovelers" to be fifteen hundred. They work in gangs of from twenty-five to fifty, under the direction of a "boss shoveler," who is usually also their landlord, deriving from this latter avocation a large part of his income, as he provides not only board and lodging, but also keeps a bar for the *accommodation* of his boarders and their friends. The meals are generally good and abundant. The sleeping apartments are close rooms and crowded lofts, with but little attention to ventilation or cleanliness. Some, however, are very much better than others in this respect. A considerable number of the men live with their families in their own tenements, but the larger proportion board as above described. The work consists in the removal of grain from vessels and cars to elevators, and from the latter to cars and canal boats. This was formerly done by hand-shoveling alone, but latterly the steam shovel, a most ingenious labor-saving invention, is generally employed, but requiring in its action a large amount of accessory work by the hand-shovel; as many as fifty men often laboring at once, in connection with it in the hold of a large propeller. Inquiries have failed to elicit the exact temperature sometimes undergone in this work, but an intelligent foreman of one of the largest elevators said it was often very high, especially when the large overlying boilers were full of very hot water, as was generally the case. Besides temperature, absence of fresh air, and dirt and dust and minute particles of grain, especially when bearded, are to be considered as factors of disease. But these are as nothing, and perhaps unavoidable, as compared with the great destructive feature now to be mentioned, and which must strike with surprise and awe, and even horror, those unfamiliar with it, viz., the often long-continued, unintermittent time of work. As to this, it is but fair to state that employers and foremen deny the statements made by the employees. The former assert that no gang ever works continuously for more than thirty-six hours, and that in some of the largest elevators never more than twelve hours, while the workmen have again and again, assured the writer with the most solemn assurances, that they have toiled consecutively for six and seven days and nights, stopping only very briefly for meals and drinks, and snatching now and then from five to ten minutes of sleep between the arrival and departure of cargoes. If this assertion is true, it is a much greater test of physical endurance than any of the shameful pedestrian exploits now so much in vogue, which really are catch-penny exhibitions, alike pernicious to the walkers and the lookers-on, and a disgrace to humanity and civilization. The truth probably is, that the shovelers occasionally exceed the longest time admitted by their employers, and rarely, if ever, attain that which they most persistently assert. To the inquiry, What do your men drink? the answer was very prompt and decided: "Mostly whisky, and when they can get it, ten or twelve times a day—some take only beer, and a few only tea or coffee." Which do the best? "The latter, and then the beer-drinkers; the whisky men do the worst."

The work is singularly intermittent. In busy seasons even, some gangs will be several days without anything to do, and a large portion of this idle time is spent in drinking and in various dissipations, and therefore it is, when a job is secured, it is pushed. The wages, for mere manual labor, are high, being so much per thousand bushels, generally $3.75 to $4.00. At these prices, a few days of hard work put a considerable amount into the pocket of the "scooper." He is, however, with but few exceptions, improvident; and a short number of idle days exhausts his store. Some, indeed, lay up for the coming winter, especially those with families, but the proportion of those who do this is lamentably small. With but few exceptions the men are Irish, many of them young and but recently arrived, and this fact is to be borne in mind when we consider that the Irish in this country (and especially the young), and perhaps also at home, are very prone to lung affections. There are a few Americans of Irish parentage, a few Germans, and occasionally a representation of other nationalities. Soon after a new man commences work, he experiences catarrhal, nasal and throat irritation, and he may labor through a whole season with nothing more than this. He is apt, however, to develop bronchitis, involving the larger, and eventually the smaller tube, and bronchioles. It is generally of a sub-acute character, rarely eventuating in capillary bronchitis, and is unattended with febrile movement or other indication of active inflammatory action. It is chiefly important to consider this condition with reference to its ultimate results, it being the ground-work for subsequent serious disease. It occasionally happens, however, that more dangerous disorder is developed after short exposure, in the form of active broncho-pneumonia, and this particularly where there is any tubercular proclivity. The second and third years are, however, fruitful of morbid effects, not uninfluenced by the idle and dissipated life of the intervening winter. It is then that the hospital becomes a necessity—and there is found every grade of a peculiar class of pulmonary disorder, ranging from uncomplicated bronchitis to broncho-pneumonia, with a considerable amount of pleuritic complication, and these associated almost invariably with great disturbance of the renal, hepatic and digestive functions. Both lungs are usually implicated. Pure pneumonia is rarely met with, but there is a great deal of hypostotis, and a semi-solidification of the posterior portions of both lungs is common, generally more extensive in the right than in the left. The pulse as a rule is frequent and feeble, the heart beats tumultuously rather than strongly, except where it is hypertrophied, which is quite often found to be the case. The skin is usually moist, sometimes as much so as in delirium tremens, of which it often reminds the examiner. The temperature varies considerably, averaging about 103°, with very little diurnal range as respects morning and evening. It is sometimes as high as 106°, and in others does not rise over 101°. In the more severe cases delirium, particularly nocturnal, is often manifested. It is usually low and muttering, but occasional instances of the mania a potu type are encountered, giving rise to a suspicion at least of delirium tremens complication. Generally there is great dyspnœa and a good deal of cough. The expectoration varies from slight muco-purulent to rusty and hemorrhagic, and is often copiously purulent and fetidly offensive, the breath giving off the same odor. From first attacks recovery, at least partial, is the rule, but convalescence is very slow, and is often interrupted by recurrence to some extent of serious symptoms, made manifest after slight exposure or indiscretion, or coming on spontaneously without being attributable to any exciting cause, and probably proceeding from new local pulmonary infiltration engorgement or softening. An attack of what is called in the hospitals elevator pneumonia usually ensures, for the subject, a ward residence of at least three months. Such persons are always advised to find some new field of labor, and if they do so not infrequently recover entirely, becoming robust and vigorous; but unfortunately they sometimes can find no new work to do, and are compelled to do what they can, while others, regardless of consequences, or skeptical as to warnings, attracted by the fellowship of gang-work, and by the lure of large wages, go back to their haunts of work and of dissipation, and soon fall victims to acute or chronic pulmonary disorder. Many of them have all the general symptoms of pulmonary phthisis, such as cough, purulent expectoration, emaciation and night sweats; but physical exploration fails to detect the true tubercular lesions, except in a few. In these, however, its progress is rapid, owing to the superinduced broncho-pneumonia and its influence in hastening destructive tubercular changes.

Post mortem appearances. Although the mortality is considerable, no very larger number of pathological observations have been made. Where death occurs at the home or boarding place it is impossible to obtain an examination, and nearly as difficult at the hospital, relatives, friends and especially the working associates, always claiming and carrying away the body soon after its demise. A continuous hospital service of twenty-six years has afforded a few post mortems. One or both pleuræ are generally adherent, and are red and thickened with more or less of false membrane. It is rare to meet with pleuritic effusion. The lungs are red, heavy and engorged in their lower and posterior portions, also soft and pulpy, and on being cut across exude frothy, bloody, creamy and purulent fluids. Occasionally deep blood-red infarctions in considerable number are found, and still more rarely an abscess of variable size, with now and then a considerable number of small pits of pus, the apparent effect of scattered spots of lobular pneumonia. The lining membrane of the larger bronchial tubes is, without exception, swollen and thickened, and covered with a thick tenacious muco-purulent secretion—this is also found in the trachea and larynx. The pericardium usually contains from one to three ounces of serum, often blood-stained, and the heart is pale, flabby and softened, and generally enlarged. In many instances fatty degeneration of liver or kidneys, or both, is noted, and it is not unusual to find considerable fluid in the abdominal cavity. No special lesions are detected in the intestinal tract, but the bowels very often contain a noticeable number of lumbricoides.

### TREATMENT.

The most dependence is placed upon the change effected by removal to the hospital, cleanliness of body and bed, enlarged and purer air space, although, in a ward, abundance of concentrated nutritments, with pure milk ad libitum, with alcoholic stimulants as required, sometimes considerable being necessary, but usually only a small amount and for a few days; quinine, according to temperature and effect; carbonate of ammonia, freely in almost every case; opium, mostly in the shape of Dover's powder, in small doses, but avoided where there is great dyspnœa with lividity of surface; tr. of chloride of iron, especially where the breath and expectoration are offensive, appears to be of great service; it is often advantageously combined with chlorate of potash. Sinapisms and large hot poultices, the latter surrounding the chest, are the only external applications. They seem to be very effectual in relieving pain and distressed breathing. Many a patient nearly moribund has appeared to be rescued by the large hot jacket. Strychnine, camphor, and the ethers are found useful when the heart needs sustaining, and this is very often the case. This is indicated by feebleness of impulse and by weakness of first sound. Daily cardiac auscultation should never be omitted; prognostically it is more important than any other examination. This paper will close with a few further remarks, the first one certainly startling. The average duration of the "scoopers" life is five years. It has been computed as low as three. It is true that there are men who year after year follow this labor until they become too old to pursue it further, but these are very rare. It is noticeable that those who commence it at thirty-five or forty years of age bear it much better than those who begin at twenty. This is probably explicable on the hypothesis that it is the healthiest period of human life, and that at this time the body is much less susceptible to morbific impressions than at earlier and later epochs of existence. That the average mortality has not been overstated, reference is made to a very recent speech of J. H. Dormer, Esq., long doing business in the neighborhood of the elevators, and made still more practically familiar with the subject by his long, earnest and devoted labor in connection with the Charity Organization. In advocating the establishment of a creche, he especially emphasized its great need for the relief and aid it would give to that most numerous class, the widows and orphans of the elevator shovelers. Our duty as medical men is plain. We have in our midst a body of laborers, who, by their work, contribute largely to our commercial prosperity, but at a terrific sacrifice of health and life.

The remedy seems to be plain and simple. Does it not consist in regular and shorter hours of continuous labor, in a sanitary regulation of lodging and boarding houses, and in as far as possible a limitation in the use of ardent spirits? This, on proper representation, can be accomplished as to hours of work by the owners of elevators, as to lodging and boarding houses by the keepers of the same, in co-operation with the municipal Board of Health. The whisky problem will be the most difficult to solve; it could be rendered much easier if the bars of boarding houses could be closed. But this would be very difficult to accomplish. The subject has been laid before you, and the writer will be most happy to hear your discussion upon it, and to co-operate most heartily in any feasible plan for diminishing the evils to which your attention has been called.

